# Tumor Necrosis Factor Inhibitor-Induced Eczematous Eruption: A Report of Two Cases and Review of the Literature

**DOI:** 10.7759/cureus.15078

**Published:** 2021-05-17

**Authors:** Sohemi Pagan-Leon, Tyler Werbel, Marjorie Montanez-Wiscovich

**Affiliations:** 1 Dermatology, School of Medicine, Ponce Health Sciences University, Ponce, USA; 2 Dermatology, University of Florida College of Medicine, Gainesville, USA

**Keywords:** tnf, th1, th2, atopic dermatitis, eczematous eruption, phenotype switch

## Abstract

Tumor necrosis factor-alpha (TNF-α) inhibitors are frequently used for the management of type 1 helper T-cell (Th1) immune-mediated chronic inflammatory conditions such as psoriasis and Crohn’s disease. Although TNF-α inhibitors are usually well-tolerated, various cutaneous side effects are frequently observed, including eczematous or atopic dermatitis-like eruptions. It is postulated that the attenuation of the Th1 immune pathway with TNF-α inhibition causes a shift towards a type 2 helper T-cell (Th2) immune response, leading to the development of skin lesions grossly and histologically consistent with the Th2 mediated disease atopic dermatitis. Herein, we describe the development of an eczematous eruption in two patients with a history of Th1-mediated disease after months of therapy with a TNF-α inhibitor.

## Introduction

An eczematous or atopic dermatitis-like eruption is a common adverse cutaneous event in patients receiving tumor necrosis factor-alpha (TNF-α) inhibitor therapy [1]. Treatment-associated eczematous skin eruption has been observed with infliximab [1], etanercept [2], and adalimumab [3]. We describe a woman who developed dyshidrotic hand dermatitis following psoriasis treatment with adalimumab and a girl who presented with an atopic dermatitis-like eruption on her forearms and neck after starting adalimumab therapy for Crohn’s disease.

## Case presentation

Case 1

A 64-year-old woman was seen for treatment of chronic plaque and inverse psoriasis with 15% body surface area involvement. The patient had a five-decade history of psoriasis and had failed multiple therapies, including methotrexate, clobetasol, fluocinonide, tacrolimus, narrow band UVB phototherapy, and tar. Her pertinent past medical history included seasonal allergies and adult-onset asthma. She had no personal history of atopic dermatitis nor family history of atopy.

**Figure 1:**
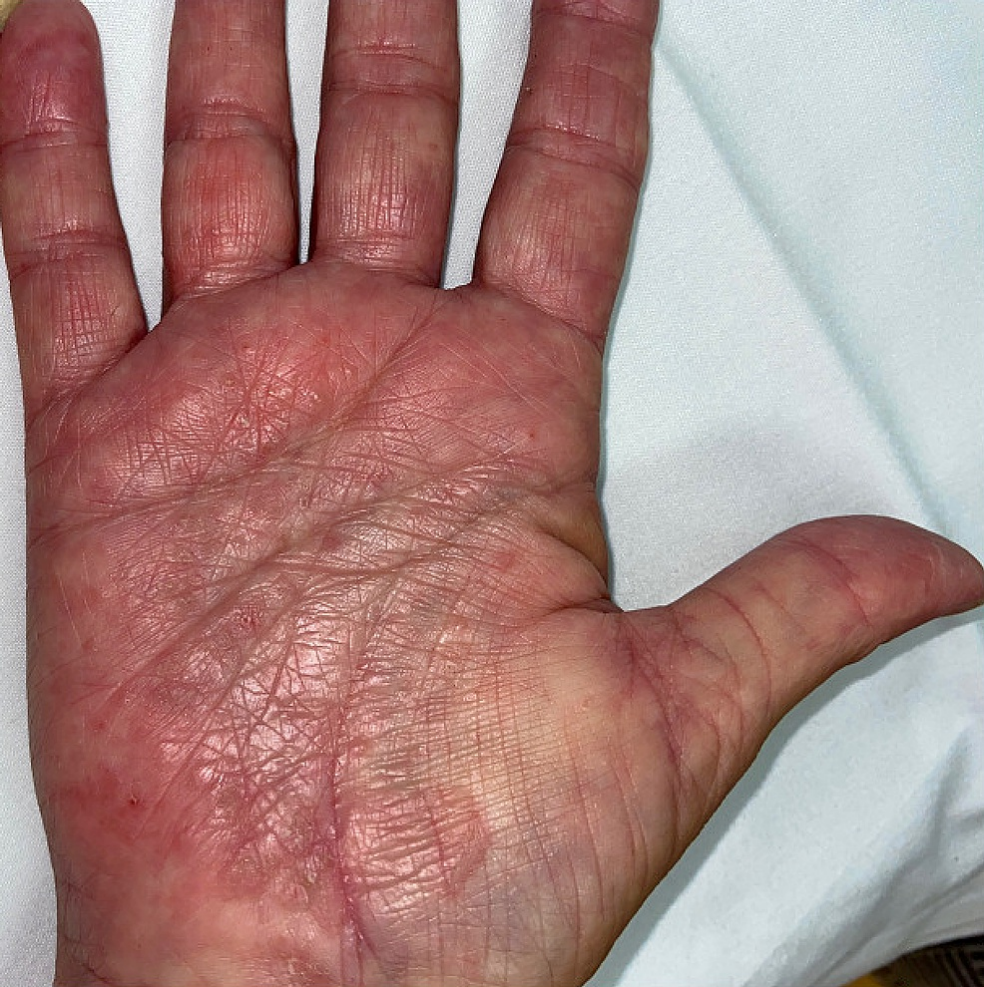
Patient 1’s ventral hands showing deep-seated vesicles within pink plaques.

The patient was started on 40 mg adalimumab biweekly after an 80 mg initial dose, which resulted in complete control of her psoriasis. After four months of therapy, she developed a pruritic vesicular rash on her hands. She denied changes to personal care products used. Cutaneous examination revealed deep-seated vesicles within pink plaques with a fine-scale involving the palmar hands bilaterally (Figure 1).

**Figure 2:**
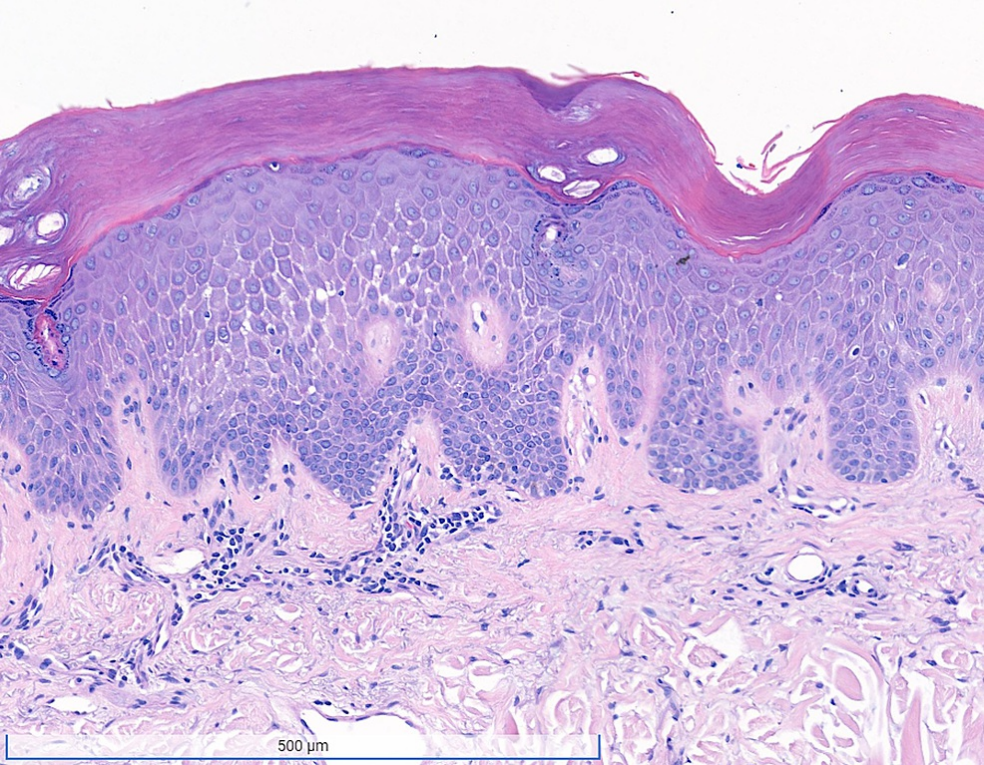
Histopathology of the skin biopsy. A 3 mm x 3 mm x 2 mm punch biopsy was performed. It revealed mild acanthosis, spongiosis and focal lymphocytic exocytosis. A sparse perivascular lymphocytic infiltrate was noted in the superficial dermis. No eosinophils were identified. (hematoxylin & eosin, x6 magnification).

A punch biopsy of the left hypothenar skin showed a mildly acanthotic epidermis with spongiosis and focal lymphocytic exocytosis. Within the superficial dermis, a sparse perivascular lymphocytic infiltrate was noted without eosinophils (Figure 2). Periodic acid-Schiff stain was negative.

Clinical and histologic findings supported the diagnosis of dyshidrotic hand dermatitis. Adalimumab therapy was not discontinued, and the patient was started on halobetasol 0.05% ointment twice daily. The hand rash resolved after two weeks of topical corticosteroids but flared after she discontinued medication.

Case 2

A 14-year-old girl presented for evaluation of a rash on the neck and arms. The patient was diagnosed with Crohn’s disease seven months prior and started treatment with adalimumab and methotrexate. After two months of therapy, she developed a pruritic rash on the dorsal neck and bilateral upper extremities that was not relieved by over-the-counter lotions and baby oil. Self-discontinuation of adalimumab and methotrexate for four weeks resulted in no significant improvement of her symptoms. Therefore, she resumed therapy with both medications before visiting our dermatology clinic. The patient has no personal history of atopy, but family history is notable for atopic dermatitis in the mother and a sibling.

**Figure 3:**
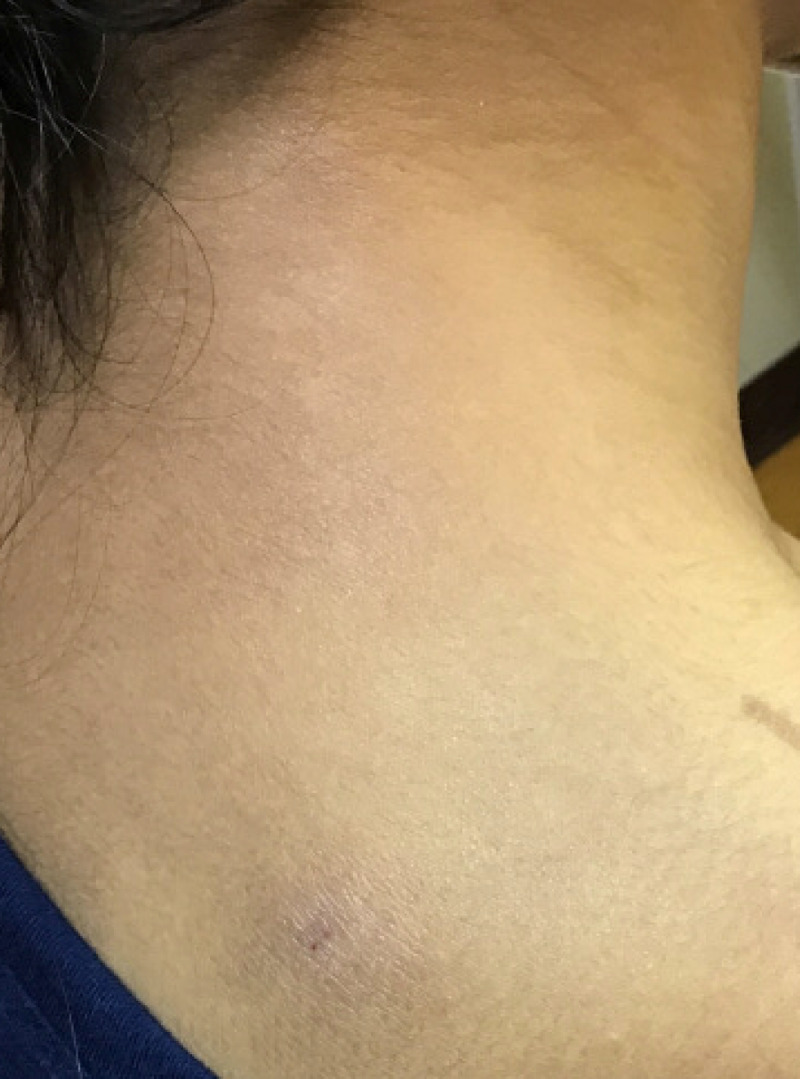
Patient 2’s eczematous eruption on the nape of the neck.

Skin examination demonstrated ill-defined erythematous scaly thin plaques and hypopigmented patches on the bilateral ventral forearms, antecubital fossae, upper arms, and nape of the neck (Figure 3). A clinical diagnosis of mild atopic dermatitis was made. Adalimumab and methotrexate were continued, and mometasone 0.1% ointment was applied twice daily to affected areas of skin with complete resolution of the rash. 

## Discussion

Atopic dermatitis and psoriasis are chronic inflammatory skin conditions driven by opposing helper T-cell populations [4]. Whereas atopic dermatitis results from a Th2-mediated immune response characterized by a cytokine signature of interleukin (IL)-4, IL-13, and IL-31, psoriasis is dominated by Th1 cells, which secrete interferon (IFN)-γ and TNF-α, and by Th17 cells, which release IL-17 and IL-22 [5,6]. Th1 cells and TNF-α have similarly been implicated in the development of intestinal inflammation in Crohn’s disease [7].

The recognition of TNF-α as a key promoter of systemic inflammation has led to the use of TNF-α inhibitors, such as infliximab, etanercept, and adalimumab in the treatment of psoriasis and Crohn’s disease [6,8]. However, it is hypothesized that the suppression of the Th1 pathway through TNF-α blockade may create an imbalance in the immune system, favoring increased activity in the antagonistic Th2 axis associated with conditions such as atopic dermatitis, allergic rhinitis, and asthma [4].

Like the patients in this report, the development of an eczematous or atopic dermatitis-like eruption following TNF-α inhibitor therapy is well-documented in multiple case reports [1,3], prospective studies [9,10], and one retrospective cohort [11] and one review [12]. In most cases, the eczematous eruption was of moderate severity and was able to be managed with topical corticosteroids while continuing TNF-α inhibition [13]. In one study, a personal history of atopic symptoms was associated with a higher rate of developing a TNF-α inhibitor-induced eczematous eruption with an odds ratio of 3.6 [10].

Interestingly, a contrasting phenomenon has been reported where patients with atopic dermatitis developed psoriasiform eruptions after treatment with dupilumab, a monoclonal antibody against Th2 cytokines IL-4 and IL-13 [14-17]. In these cases, it is postulated that the inhibition of the Th2 pathway by dupilumab leads to an activation of the opposing Th1 axis, manifesting as psoriasis. Although most reports of dupilumab-induced psoriasiform eruptions lack a personal or family history of psoriasis, one small case series noted a more severe presentation in one patient with a family history of psoriasis. It is possible that a genetic predisposition may lead to a more prominent Th2 to Th1 switch [18]. Further investigation is needed to clarify this association.

## Conclusions

In summary, physicians should be aware that TNF-α inhibitors may cause an eczematous eruption as a result of a Th1 to Th2 phenotype switch. In particular, patients with a strong personal or family history of atopy should be advised of this potential side effect. Typically, the eczematous eruption can be managed with topical corticosteroids without discontinuing TNF-α inhibitor therapy.
